# False Report

**Published:** 1881-12-20

**Authors:** 


A FALSE REPORT.—The very circumstantial report which we published as it went the rounds of the press, giving the fees of the medical attendants of the late President, turns out to be without foundation. It is denied by authority, and the assertion positively mede that no bills will be presented. An *honorarium* is expected from Congress when it meets, though delicacy restrains the doctors from presenting an account for their certainly very laborious services. They may rest assured that posterity will say they deserved well of their country.—*Louisville Medical News.*

